# The Anti-Inflammatory, Phytoestrogenic, and Antioxidative Role of *Labisia pumila* in Prevention of Postmenopausal Osteoporosis

**DOI:** 10.1155/2012/706905

**Published:** 2012-03-15

**Authors:** M. E. Nadia, A. S. Nazrun, M. Norazlina, N. M. Isa, M. Norliza, S. Ima Nirwana

**Affiliations:** Department of Pharmacology, Faculty of Medicine, The National University of Malaysia, Kuala Lumpur campus, 50300 Kuala Lumpur, Malaysia

## Abstract

Osteoporosis is characterized by skeletal degeneration with low bone mass and destruction of microarchitecture of bone tissue which is attributed to various factors including inflammation. Women are more likely to develop osteoporosis than men due to reduction in estrogen during menopause which leads to decline in bone-formation and increase in bone-resorption activity. Estrogen is able to suppress production of proinflammatory cytokines such as IL-1, IL-6, IL-7, and TNF-*α*. This is why these cytokines are elevated in postmenopausal women. Studies have shown that estrogen reduction is able to stimulate focal inflammation in bone. *Labisia pumila* (LP) which is known to exert phytoestrogenic effect can be used as an alternative to ERT which can produce positive effects on bone without causing side effects. LP contains antioxidant as well as exerting anti-inflammatory effect which can act as free radical scavenger, thus inhibiting TNF-*α* production and COX-2 expression which leads to decline in RANKL expression, resulting in reduction in osteoclast activity which consequently reduces bone loss. Hence, it is the phytoestrogenic, anti-inflammatory, and antioxidative properties that make LP an effective agent against osteoporosis.

## 1. Introduction

 Plant has been one of the sources of medicine to treat various illnesses and diseases since ancient time. In the early 19th century, when chemical analysis first became available, scientists began to extract and modify the active ingredients from plants which later led to wide development of natural or traditional medicine that was mostly passed on orally from one generation to another. More than 35,000 plant species have been reported to be used in various human cultures around the world for their medical purposes [1]. Traditional medicine has been defined by the World Health Organization (WHO) as “health practices, approaches, knowledge and beliefs incorporating plant, animal and mineral-based medicines, spiritual therapies, manual techniques and exercises, applied singularly or in combination, to treat, diagnose and prevent illnesses or maintain well-being” [2].

 Currently in Malaysia, over 2,000 species of lower plants with medicinal and therapeutic properties have been identified, and most of them have been used for many generations in various health care systems. About 17.1% of Malaysians used herbs to treat their health problems while 29.6% of them consumed herbs for their health maintenance [3]. The earliest report on medicinal plant research in Malaysia was carried out by Arthur in 1954 [4]. Subsequently, more plants were screened chemically for alkaloids, saponins, triterpenes, and steroids in the 90s [5, 6]. 

 Amongst the famous herbs that are widely used in Malaysia by the locals are *Labisia pumila* (Kacip Fatimah), *Eurycoma longifolia Jack* (Tongkat Ali), *Orthosiphon stamineus* (Misai Kucing), *Quercus infectoria* (Manjakani), and *Piper sarmentosum* (daun kaduk). These plants are similar in terms of exhibiting phytochemical properties that are protective against various diseases. These herbs are known to exert antibacterial, antioxidant, and anti-inflammatory properties that make them beneficial against many types of diseases such as fever, asthma, joint pains, gastrointestinal diseases, bone disorders, and inflammatory disorders. [7–9]. This paper is a review which will be focusing on the content and health benefits of one of the famous Malaysian herbs, Kacip Fatimah.

 Kacip Fatimah or its scientific name *Labisia pumila *(LP) is a member of small genus of slightly woody plants of the family Myrsinaceae. There are four known varieties of *Labisia pumila* found in Malaysia but only three of them are widely used by the locals, which are recognized as *Labisia pumila *var. *pumila, Labisia pumila *var. *alata,* and *Labisia pumila *var. *lanceolata* [10, 11]. LP is found mainly in the lowland and hillforests of peninsular Malaysia at an altitude between 300 and 700 metres. It is also known by the locals as Selusuh Fatimah, Rumput Siti Fatimah, Akar Fatimah, Pokok Pinggang, and Belangkas Hutan [12, 13]. Of all the subtypes, *Labisia pumila *var. *alata* is the most widely used by the locals [10]. Its water extract is traditionally consumed especially by the Malay women to treat menstrual irregularities and painful menstruation, help contracting birth channel after delivery, and to promote sexual health function [14, 15]. It has also been used to treat dysentery, gonorrhoea, rheumatism, and sickness in bones [16, 17].

 It is the phytoestrogen, anti-inflammatory, and antioxidative properties that make LP effective against various illnesses. LP was reported to exert estrogenic properties [18–20]. Theoretically, phytoestrogens can act as anti-estrogenic agents by blocking the estrogen receptors and exerting weaker estrogenic effect compared with the hormone [21]. The water extract of LP has been found to inhibit estradiol binding to antibodies raised against estradiol, suggesting the presence of estrogen-like compounds in the extract [22]. It also contains triterpene and saponins, including the compound ardisiacrispin A which were thought to be the reason behind the phytoestrogenic activity of LP [23].

 LP has been widely used by the locals in Malaysia not only to ease menstrual pain, induce labor, and promote healthy sexual function but it is also used as an alternative to estrogen replacement therapy in postmenopausal women [24, 25]. Postmenopausal women are prone to osteoporosis due to the reduction in estrogen level. Estrogen acts on estrogen receptor-*α* (ER*α*) and receptor-*β* (ER*β*) which has high affinity towards osteoblasts and osteoclasts [26]. Activation of estrogen-receptor complex is vital in maintaining bone remodelling processes [27]. Estrogen can induce osteoclasts apoptosis and inhibit osteoblasts apoptosis, which indirectly will reduce bone resorption and increase bone-formation activity [28]. Hence, reduction in estrogen is highly associated with bone loss. Dietary phytoestrogens such as LP can be an alternative to synthetic estrogen for hormone therapy to reduce side effects of prolonged hormone therapy such as risk of breast cancer, endometrial cancer, and cardiovascular diseases [29, 30]. This paper will focus on the role of *Labisia pumila* in offering protection against postmenopausal osteoporosis via its anti-inflammatory properties.

## 2. Anti-Inflammatory Role of *Labisia pumila*

Osteoporosis is characterized by skeletal degeneration with low bone mass and destruction of microarchitecture of bone tissue. According to the National Institute of Health, osteoporosis is a skeletal disease which involves decline in mass and density which later leads to fracture [31]. Women, especially postmenopausal women, are more likely to develop osteoporosis than men due to tremendous decline in estrogen during menopause which will lead to decline in bone formation and increase in bone-resorption activity [32]. Osteoporosis is attributed to various factors, and there are evidences that inflammation also exerts significant influence on bone turnover, inducing osteoporosis [33, 34]. According to studies by Lorenzo and Manolagas and Jilka, certain pro-inflammatory cytokines play potential critical roles both in the normal bone remodeling process and in the pathogenesis of osteoporosis [34, 35]. For example, interleukin- (IL-) 6 promotes osteoclasts differentiation and activation [36]. IL-1 is another potent stimulator of bone resorption [37] that has been linked to the accelerated bone loss seen in postmenopausal osteoporosis [38].

 Various epidemiologic studies reported an increase in the risk of developing osteoporosis in various inflammatory conditions such as rheumatoid arthritis, haematological diseases, and inflammatory bowel disease [39, 40]. Proinflammatory cytokines such as tumor necrosis factor (TNF)-*α*, IL-6, IL-1, IL-11, IL-15, and IL-17 are elevated in these conditions [41]. IL-6 and IL-1 may influence osteoclastogenesis by stimulating self-renewal and inhibiting the apoptosis of osteoclasts progenitors [42, 43]. They promote osteoclasts differentiation which is an important stimulator of bone resorption that has been linked to accelerated bone loss seen in postmenopausal women [36]. Receptor activator of NF-*κβ* ligand (RANKL) is a membrane-bound molecule of TNF ligand family which plays a crucial role in osteoclasts formation [44]. TNF is a cytokine that is involved in inflammation and is an important cofactor in bone resorption because this cytokine supports osteoclasts activation mediated by RANKL and c-Fms/macrophage colony-stimulating factor.

 Estrogen is able to suppress the production of these proinflammatory cytokines [45, 46]. This is why estrogen withdrawal following menopause will lead to increase in these cytokines as proven in many studies. Studies on bone resorption demonstrated that the fall of estrogen level in postmenopausal women was able to stimulate local inflammation in the bone. Ovariectomy in rats was accompanied by increased production of IL-1 and TNF-*α* which later resulted in decrease in bone density. Hence, it is suggested that estrogen withdrawal can be associated with an increase in production of proinflammatory cytokines, which in turn increases osteoclasts activity resulting in profound bone loss [47]. Estrogen will stimulate production of osteoprotegerin (OPG), which is a potent antiosteoclastogenic factor. OPG acts as a decoy, blocking the binding of the RANK expressed in osteoblasts progenitors, to RANKL which is expressed in committed preosteoblastic cells [48]. This estrogen deficiency leads to upregulation of cytokines [49] and down-regulation of OPG which will result in increase in inflammatory responses and increase in bone-resorption activity. In a study by Collin-Osdoby et al., [50] increases in RANKL and OPG mRNA expression were seen in endothelial cells following an inflammatory stimulus. Therefore, suppression of these potent inflammatory mediators has been proposed to explain the deleterious effects of estrogen deficiency on the human skeletal system at menopause.

## 3. Phytoestrogenic Role of *Labisia pumila*

 LP which has been opposed to exert phytoestrogen property can be used as an alternative to estrogen replacement therapy (ERT) in postmenopausal inflammation-induced osteoporosis. In contrast to ERT which can cause many harmful side effects, LP which originated from natural resources will not cause any side effect, if taken within its safe therapeutic dose. Toxicity testing of LP which was done by the Herbal Medicine Research Centre of Institute of Medical Research has shown that LD50 is safe at more than 5.0 g/kg [51]. LP extract was found to exhibit no-adverse-effect level (NOAEL) at the dose of 50 mg/kg in subacute toxicity study [52], 1000 mg/kg in subchronic toxicity study [53], and 800 mg/kg in reproductive toxicity study [51]. Therefore, LP is safe to be given at high dose as long as it does not outweigh the toxic dose.

 Studies have shown that production of proinflammatory cytokines in response to estrogen withdrawal at menopause is responsible to the stimulation of osteoclastic bone resorption [54–56]. A study done by Choi et al. [57] indicated that the LP extract may have good potential to be developed as novel anti-inflammatory drug due to an experimental finding of treatment with LP extract which has markedly inhibited the TNF-*α* production and the expression of cyclooxygenase (COX)-2. COX-2 is an enzyme that is responsible for the production of mediators involved in inflammation. In vitro experiments have revealed increased COX-2 expression after stimulation with proinflammatory cytokines, such as IL-1 and TNF-*α* [58].

 Pharmacological inhibition of COX can provide a relief from the symptoms of inflammation and pain. Studies have shown that COX-2 plays an important role in pathophysiology of osteoporosis by stimulating the production of prostaglandin (PGE_2_). Excessive PGE_2_ production might lead to increase in bone resorption, while deficient of its production might impair the bone-formation response, both to mechanical loading and remodelling [59]. Consequently, inhibition of the COX-2 enzyme in postmenopausal women may prevent menopausal bone loss [60]. Inhibition of the main proinflammatory cytokines has proven that LP extract could be a good material for the regulation of anti-inflammation process. TNF has been shown to stimulate osteoclast differentiation, increase its activation, inhibit its apoptosis, and inhibit osteoblast differentiation [61–63]. It also reduces bone formation in cultured osteoblast *in vitro* [64]. Similar to IL-1, TNF-stimulated induction of osteoclast-like-cell formation in bone marrow culture is mediated by increases in RANKL expression. However, in addition to increasing RANKL expression, TNF also inhibits OPG in an osteoblastic model [65]. Hence, inhibition of TNF will indirectly help in reducing bone loss.

## 4. Antioxidative Role of *Labisia pumila*

 Based on previous studies, LP has been shown to exhibit antioxidative properties due to the presence of flavanoids, ascorbic acid, beta-carotene, anthocyanin, and phenolic compounds [66, 67]. According to Norhaiza et al. [68], there were positive correlations between the antioxidant capacities and the antioxidant compounds of LP extract with *β*-carotene having the best correlation, followed by flavonoid, ascorbic acid, anthocyanin, and phenolic content. *β*-carotene is one of the basic constituent of antioxidative effect. The chemical abilities of *β*-carotene to quench singlet oxygen and to inhibit peroxyl free radical actions are well established [69]. Flavonoid has been shown to be highly effective scavenger of free radicals that are involved in diseases such as osteoporosis and rheumatism which is associated with aging due to oxidative stress [70]. Anthocyanin and phenolic on the other hand, not only play a role as antioxidative agents, but also as anti-inflammatory agents [71–73]. These antioxidative and anti-inflammatory properties of LP extract explained the effectiveness of this medicinal plant against various diseases such as osteoporosis, rheumatism, and women sexual function.

 Osteoporosis in postmenopausal women can also be explained in terms of oxidative stress mechanism. Ovariectomy has been proposed by many studies as a model of postmenopausal osteoporosis. Following ovariectomy, decline in estrogen level will result in significant bone loss due to bone resorption outweighing bone-formation activity [74]. Estrogen can be considered as an antioxidant as it was found to exhibit antioxidant protection of lipoproteins in the aqueous system [75] and was also shown to increase the expression of glutathione peroxidase in osteoclasts [76]. That is why decline in estrogen will lead to increase in osteoclasts activity resulting in bone loss. Free radicals are continuously produced in the body, mostly by biochemical redox reactions involving oxygen, which occur as part of normal cell metabolism. Free radicals, mainly reactive oxygen species (ROS), are efficiently scavenged, but oxidative stress occurs when there is an imbalance between increased ROS and inadequate antioxidant activity [77] which consequently accelerates aging process and leads to degenerative diseases such as osteoporosis, rheumatism, and cardiovascular disease.

 ROS alter mitochondrial and nuclear DNA integrity by increasing the risk of mutations. When DNA repair mechanisms are overwhelmed, cells undergo apoptosis which will lead to tissue damage [78]. This can be applicable in postmenopausal osteoporosis mechanism. When body is subjected to high oxidative stress following estrogen reduction, lipid accumulation will occur. Lipid peroxidation will promote osteoblast apoptosis and simultaneously upregulating ROS production [79, 80]. ROS was shown to promote osteoclast resorption activity either directly or mimicking RANK signalling and stimulating osteoclast differentiation, or indirectly, by stimulating osteoblast/osteoclast coupling and subsequent osteoclast differentiation [81]. Oxidative stress has been acceded as a major contributor to the immune response. Activation of immune response mechanism is characterized by establishment of an inflammatory response. Thus, osteoporosis can be associated with inflammatory mechanism.

 Estrogen can prevent osteoblast cell death and RANKL stimulation by suppressing ROS. Estrogen deficiency is a key step in ROS-mediated stimulation of bone loss via TNF-*α* signalling pathway. Stimulation of this proinflammatory cytokine will induce bone resorption by indirectly affecting production of essential osteoclast differentiation factor, thereby enhancing proliferation of osteoclast lineage [82]. Glutathione peroxide (GPx) and superoxide dismutase (SOD) are the main antioxidative enzymes that play a pivotal role in counteracting oxidative stress [83]. These enzymes were found to be lowered in postmenopausal women with osteoporosis. This failure of antioxidant defences will result in deleterious effect of hydrogen peroxide on bone health [84]. Studies of antioxidant supplementation such as vitamin E on postmenopausal rat model have shown that lipid peroxidation was successfully inhibited and antioxidative enzymes were restored to acceptable level. In study by Norazlina et al. (2007), IL-6 level was high in ovariectomised rats showing high bone resorption rate, and this level was significantly reduced after three months of tocotrienol (vitamin E) supplementation. In the same study, vitamin E-deficient rats given palm vitamin E showed an improvement in bone calcium content and reduced bone resorption marker [85]. Hence, it is shown that antioxidant is effective in reducing bone-resorption activity as well as improving bone calcium content.

 Main antioxidative compound in LP such as flavonoid and *β*-carotene has been shown in previous studies to inhibit production of nitric oxide and expression of inducible nitric oxide synthase (iNOS) [86] most likely by suppression of NF-*κ*B [87]. NF-kB is an oxidative stress-responsive transcription factor which is activated by free radicals, inflammatory stimuli, and other cytokines. Thus, free radicals may increase bone resorption through activation of NF-kB. It has previously been shown *in vitro* and in rodents that free radicals are involved in osteoclastogenesis and in bone resorption [88]. Oxidative stress may increase bone resorption through activation of NF-*κ*B which plays an important role in osteoclastogenesis [89, 90]. Hence, supplementation of LP which contains antioxidative properties can reduce oxidative stress level which indirectly prevents bone loss.

 According to a recent study by Nazrun et al. (2011), osteocalcin, a bone formation marker, was found to be lowered in ovariectomised rats. After being treated with LP results showed an increase in osteocalcin to the level seen in sham-operated group indicating normalisation of bone formation. Bone resorption marker, CTX on the other hand, was found to be reduced after the rats were treated with LP [91]. CTX is sensitive and specific in detection of osteoporosis [92]. This result showed that LP was as effective as estrogen in preventing changes in bone markers induced by ovariectomy.

 Based on its positive effects on the bone markers of ovariectomised rats which are comparable to estrogen and its safety profile, LP has the potential to be used as an alternative treatment for postmenopausal osteoporosis. All in all, it is the anti-inflammatory, phytoestrogenic, and antioxidative properties of LP that make it an effective natural medicine in treatment and prevention of osteoporosis.
